# A rare association - amelogenesis imperfecta, platispondyly and bicytopenia: a case report

**DOI:** 10.1186/s13256-015-0724-3

**Published:** 2015-10-28

**Authors:** Samir Laouina, Siham Chafai El Alaoui, Rachida Amezian, Abderrahmane Al Bouzidi, Abdelaziz. Sefiani, Mustapha El Alloussi

**Affiliations:** 1 Faculty of Medicine and Pharmacy Rabat, University Mohammed V Rabat, avenue Mohamed Belarbi El Alaoui, Rabat Institut, BP 6203, 10000 Rabat, Morocco; 2 Faculty of Dentistry Rabat, Centre for Dental Consultation and Treatment, Department of Pediatric Dentistry, University Mohammed V Rabat, avenue Allal el Fassi, rue Mohammed Jazoulit cité Al Irfane BP 6212 Rabat Institut, 10000 Rabat, Morocco; 3 Military Hospital Mohammed V, BP 1018 Hay Riad, 10100 Rabat, Morocco; 4 Genomic Center of Humain Sciences, Department of Medical Genetics, Institut National d’Hygiène, University Mohammed V Rabat, 27 avenue Ibn Batouta, BP 769, 10100 Rabat, Morocco

**Keywords:** Amelogenesis imperfecta, Bicytopenia, Platyspondyly

## Abstract

**Introduction:**

Amelogenesis imperfecta is an inherited disease characterized by generalized structural abnormalities of the enamel on all teeth, including both primary and permanent dentition. To the best of our knowledge, this is the first case report of a rare association of amelogenesis imperfecta, platyspondyly, and bicytopenia.

**Case presentation:**

A 5-year-old Moroccan boy was examined in the Centre for Dental Consultation and Treatment, Faculty of Dentistry, Rabat. He was a child of consanguineous parents (first degree). The child failed to thrive (−4 standard deviation score) and displayed delayed overall development. A dental examination revealed a hypoplastic amelogenesis imperfecta with a bacterial biofilm deposit on tooth surfaces. A complete blood count revealed bicytopenia (normocytic-normochromic anemia with thrombocytopenia). A radiographic examination of the spinal column showed a deviation of the spine in the frontal plane in the form of thoracolumbar scoliosis. The interpedicular distance was not expanded; but a mild platyspondyly exists, especially pronounced in T11 and T12.

**Conclusions:**

No other family members presented amelogenesis imperfecta, bicytopenia, or platyspondyly. The consanguineous marriage suggested an autosomal recessive mode of inheritance. Further studies are necessary to clarify the genetic defect producing this syndrome, and the symptomatic associations of amelogenesis imperfecta, platyspondyly and bicytopenia.

## Introduction

Amelogenesis imperfecta (AI) is a genetically and phenotypically heterogeneous group of inherited disorders that affects the quality and quantity of primary and/or permanent enamel, which may be associated with other morphologic or biochemical changes elsewhere in the body [1]. The population incidence varies from 1:700 to 1:16,000, depending on the diagnostic criteria used and the populations studied [2]. Dental problems, which vary depending on the severity of the condition, include sensitive teeth and poor tooth appearance, due to tooth loss and staining [3].

AI has been categorized into four broad groups based primarily on phenotype: hypoplastic, hypocalcified, hypomaturation, and hypomaturation-hypoplastic. At least 14 subtypes of AI also exist, when phenotype and mode of inheritance are considered [3]. AI associates with inclusions and abnormalities in dental eruption, congenitally missing teeth, anterior open bite, pulpal calcifications, dentine dysplasias, root and crown resorption, hypercementosis, root malformations, and taurodontisme [4].

Brachyolmia is characterized clinically by short trunk stature and radiographically by generalized platyspondyly, without significant epiphyseal, metaphyseal, or diaphyseal changes in long bones [5].

AI and platyspondyly have been previously reported in the literature [6, 7]. This is the first description of a Moroccan patient with a rare association of amelogenesis imperfecta, platyspondyly and bicytopenia.

## Case presentation

A 5-year-old Moroccan boy was referred to the Department of Pediatric Dentistry for dental treatment. He is the first child of a healthy consanguineous couple (first degree). Both parents have normal teeth. No dental problems were reported in other family members.

A detailed medical, dental and social history of the parents was obtained. The medical history and general physical condition were unremarkable.

The boy was born at term after a normal pregnancy, with low birth measurements (weight 1500 g, length 39 cm).

At 24 months of age, he could neither crawl nor walk. He was hospitalized several times to investigate the cause of his failure to thrive, his facial dysmorphology (a thin triangular face, almond-shaped eyes, a big nose and short neck), and swelling of his hands and feet. A physical examination at age 30 months revealed a body weight of 8000 g [−4 standard deviation score (SDS)]. His vital signs were within normal range. In general, the boy was small, thin, and hyperactive with aggressive behavior. Also he was noted to have brief episodes of loss of consciousness.

An examination of his cardiovascular, respiratory, and neurological systems was unremarkable.

Abdominal ultrasonography showed a normal hepatobiliary gallbladder without any anomaly identified.

Frontal digital radiography of his rib cage showed a normal mineralization of the bone matrix, no rib injuries, with rib number and morphology appearing normal.

The genitourinary examination revealed an intra-abdominal testicle.

A complete blood count revealed bicytopenia (normocytic-normochromic anemia with thrombocytopenia): a hemoglobin concentration of 118 g/l (range, 115–140), a hematocrit level of 34 % (range, 37–45 %), a red blood cell count of 3.7 M/μl (range, 3.2–5.5), a total white blood cell count of 9400/μl (neutrophil count 2162/μl), and a platelet count of 126,000/μl (range, 150,000–450,000). Peripheral blood smears of our patient confirmed normocytic-normochromic anemia and low platelet counts.

A cytomorphological examination showed his bone marrow to be free of blast or malignant cells, with normal maturation of the erythroid (18 %) and neutrophil (62 %) lineage. Eosinophils (2 %), lymphocytes (18 %), and megakaryocytes were also of normal number and morphology.

The metabolic workup, including tests for serum electrolytes, fasting blood glucose, renal function, aspartate aminotransferase (AST), alanine aminotransferase (ALT), iron and ferritin, were all within normal limits.

His urinalysis was normal (pH 6, protein negative, ketone negative, blood negative, leukocytes negative) and urine cultures were negative.

A complete infectious workup, including a human immunodeficiency virus (HIV) test, was negative.

The values of immunoglobulin A (IgA) and IgG antitissue transglutaminase antibodies were negative.

The thyroid function tests and the cortisol levels taken at 8:00 a.m. were within normal limits.

Results of somatomedin C [insulin-like growth factor 1 (IGF-1)] and testosterone tests were as follows: IGF-1 16 μg/l (range, 27–114) and testosterone 0.08 ng/ml (range, 1.56–8.77).

The insulin tolerance test showed no response to growth hormone (GH). Thus, the diagnosis of GH deficiency was confirmed. This deficiency was treated with injections of GH until the age of puberty.

A brain magnetic resonance imaging (MRI) scan was unremarkable.

A cytogenetic examination revealed a chromosomal formula 46, XY, a minor increase in the rate of chromosomal breaks (15 breaks), with a triradial picture.

**Fig. 1:**
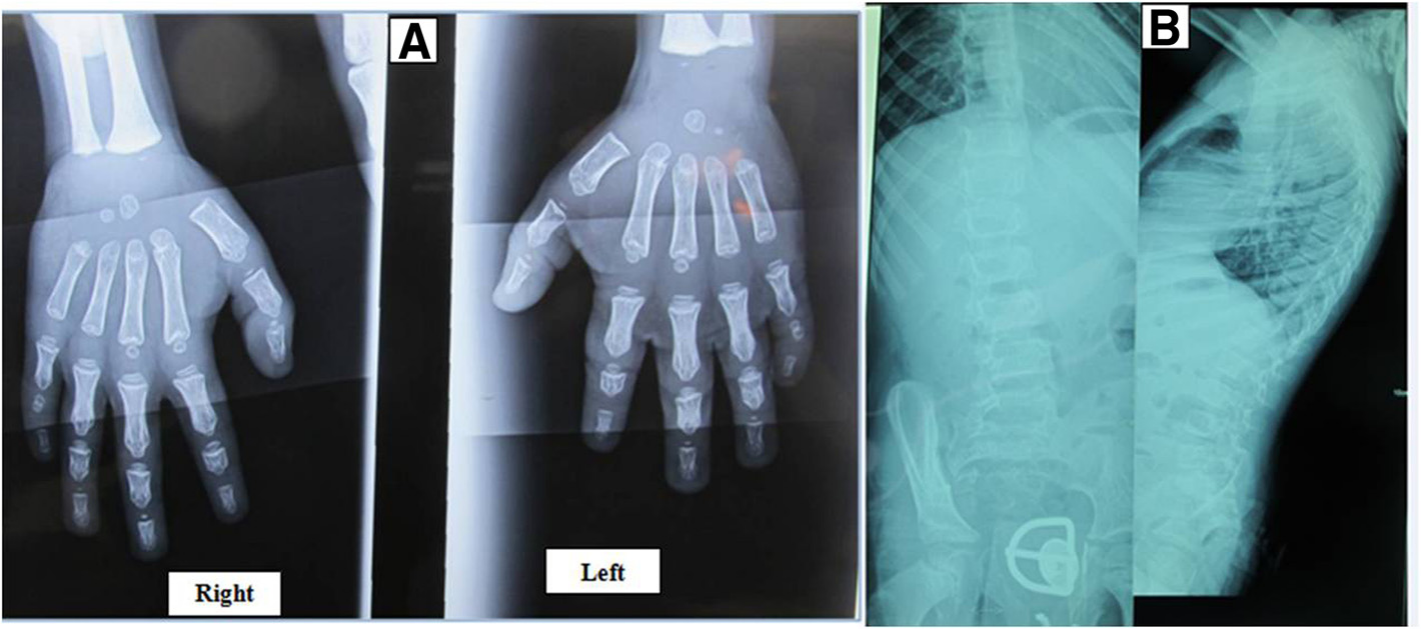
Hands-wrist and lumbar spine radiographs of the boy at the age of 30 months. **a** Hands-wrist radiograph shows normal bone structure, but bone age is delayed by 3 months for a 30-month-old child. **b** Lumbar spine radiograph shows a deviation of the spine in the frontal plane in the form of a thoracolumbar scoliosis with a mild platyspondyly

A radiographic examination of his hands showed a normal bone structure, but bone age was delayed by 3 months in this 30-month-old child. A brachymesophalangy, cone epiphyses at the second phalanges, and pseudoepiphysis at the metatarsals were observed (Fig. 1).

A radiographic examination of his spinal column showed a deviation of the spine in the frontal plane in the form of a thoracolumbar scoliosis. The interpedicular distance was not expanded; but a mild platispondyly exists, especially in T11 and T12 (Fig. 1).

**Fig. 2:**
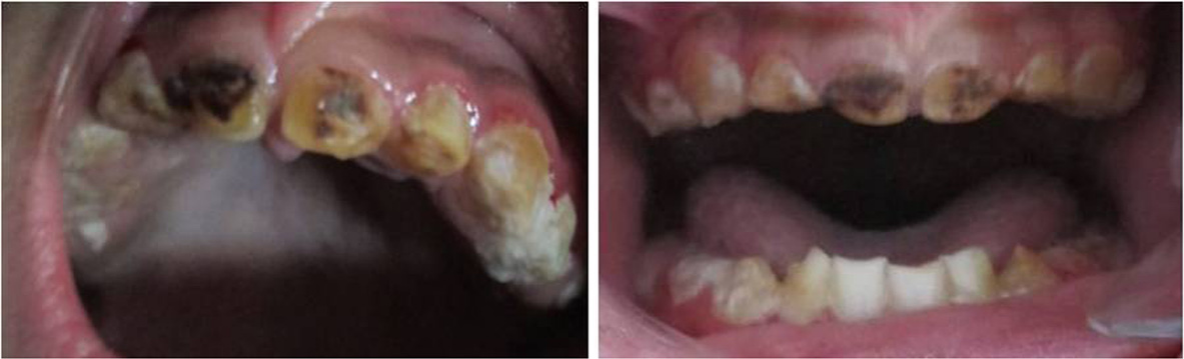
Intraoral view of the boy at 2 years old reveals hypoplastic amelogenesis imperfecta with a bacterial biofilm deposit on tooth surfaces

A dental examination revealed hypoplastic AI with a bacterial biofilm deposit on tooth surfaces (Fig. 2).

## Discussion

In 1996 Verloes *et al*. [6] described the case of a 12-year-old boy (brother) and a 16-year-old girl (sister) born of consanguineous parents with amelogenesis imperfecta and platyspondyly (OMIM 601216). At the age of 12, the boy was 133 cm tall, with a sitting height of 63 cm, and arm span of 134 cm. The girl was 141 cm tall at age 16, with an arm span of 142 cm, a sitting height of 72 cm, and short hands. The clinical features were amelogenesis imperfecta associated with short trunk and brachyolmia (that is, a generalized platyspondyly with short pedicles, narrow intervertebral and interpedicular distances, rectangular-shaped vertebrae with posterior scalloping, herniation of the nuclei, and broad femoral necks) [6].

Bertola *et al*. [7] described two other families (two brothers and one unrelated patient), presenting with amelogenesis imperfecta, brachyolmia (generalized platyspondyly), and taurodontism (OMIM 601216). In addition to that, the two brothers had congenital absence of the second mandibular premolars and hypoplastic amelogenesis imperfecta. The unrelated patient had retarded eruption of permanent teeth. The radiologic appearance of the dentition in these three cases is identical, but different from that observed in the case report of Verloes *et al*.

Verloes *et al*. and Bertola *et al*. suggested an autosomal recessive mode of inheritance [6, 7]. Mutation in the latent transforming growth factor (TGF)-beta binding protein 3 (LTBP3) gene causes brachyolmia with amelogenesis imperfecta [8].

Interestingly, in all of these cases, no hematologic abnormalities have been reported. In our case, the patient has been diagnosed clinically as bicytopenia, with GH deficiency, a mild platispondyly and hypoplastic amelogenesis imperfecta.

Hence while the combination the GH deficiency with amelogenesis imperfecta have been reported [9], the association of bicytopenia with amelogenesis imperfecta, mild platispondyly, and GH deficiency has never been observed, hence the genetic basis for this heterogeneity is to date unknown.

The bicytopenia in children can be caused by a wide variety of alterations, often leading to diagnostic uncertainty. Etiologies range from congenital and acquired bone marrow failure, to marrow space-occupying lesions, peripheral destruction of hematopoietic cells, autoimmune disorders, infection, and ineffective marrow production [10].

Inherited causes of bone marrow failure encompass Fanconi anemia, dyskeratosis congenita, Shwachman-Diamond syndrome, and congenital amegakaryocytic thrombocytopenia [10].

Fanconi anemia is characterized by bicytopenia/pancytopenia associated with multiple congenital anomalies (such as skeletal abnormalities, small stature, skin hyperpigmentation, urogenital abnormalities, and mental deficiency), high spontaneous chromosomal breakage and the tendency toward development of malignant disease [10].

In our case, the cytogenetic examination revealed a minor increase in the rate of chromosomal breaks (15 breaks), that suggested Fanconi anemia, but careful examination of clinical data, laboratory findings, and family history could not confirm this diagnosis.

Furthermore, the rate of chromosomal breakage is not sufficient to establish the diagnosis of Fanconi anemia in the absence of other phenotypic anomalies. Hence clinical findings are insufficient to determine the underlying etiology of the bicytopenia [11].

The clinical features appear to have some features of a hereditary condition of amelogenesis imperfecta in which enamel structure is defective. The mode of inheritance appeared to be autosomal recessive because of consanguinity and a negative family history for any bone or dental abnormalities.

## Conclusions

Further research is necessary to clarify the genetic defect behind this syndrome, which combines three uncommon conditions, such as amelogenesis imperfecta, platyspondyly and bicytopenia.

## Consent

Written informed consent was obtained from the patient’s family for publication of this case report and any accompanying images. A copy of the written consent is available for review by the Editor-in-Chief of this journal.
